# Machine learning-based prediction of pervaporation permeation using physicochemical properties of permeant-membrane and process conditions

**DOI:** 10.1016/j.heliyon.2025.e42714

**Published:** 2025-02-13

**Authors:** Muhammad Mujiburohman, Marwen Elkamel, Farzad Hourfar, Ali Elkamel

**Affiliations:** a Chemical Engineering Department, Universitas Muhammadiyah Surakarta (UMS), 57102, Indonesia; b Industrial Engineering & Management Systems Department, University of Central Florida, Orlando, FL, 32816, USA; c Department of Chemical and Materials Engineering, University of Alberta, Edmonton, Alberta, Canada; d Department of Chemical Engineering, University of Waterloo, Waterloo, Ontario, Canada; e Research and Innovation Center on CO_2_ and Hydrogen (RICH Center) and Chemical and Petroleum Engineering Department, Khalifa University of Science and Technology, Abu Dhabi PO Box 127788, United Arab Emirates

**Keywords:** Artificial neural network, Machine learning, Pervaporation flux, Physicochemical properties, Process conditions

## Abstract

It is well accepted that the pervaporation (PV) permeation was affected by the physicochemical properties of permeant-membrane materials and process conditions. Given many experimental data of PV, a predictive model on PV permeation based on their physicochemical properties and process conditions can be constructed. This study proposes a machine learning approach in terms of artificial neural network (ANN) to predict the permeation flux of PV, as a function of physicochemical properties of permeant-membrane material and process conditions. A large dataset was assembled from the literature and was divided into a training subset and a testing subset. The output variable was the PV flux, while the input variables were the physicochemical properties of permeant-membrane and the process conditions which were considered to affect the PV flux. Two types of inputs were evaluated: regular variables (Type I) and dimensionless groups derived from these regular variables (Type II). Several neural network architectures were evaluated. The best predictive performance for Type I inputs was achieved with a deep neural network consisting of two layers, each with 7 neurons. For Type II inputs, the optimal architecture was a shallow neural network with a single layer containing 6 neurons. The correlation coefficients (*R*) during model training for Type I and Type II were 0.93674 and 0.88332, respectively, while the root mean square errors (RMSE) were 42.2558 and 38.0766, respectively. The extent of dependency of output on input variables was determined using Garson's equation. It was found that the most affecting physicochemical properties on the PV permeation flux were glass transition temperature (*T*_*gm*_), solubility difference of two different permeants $\left(\Delta Sol_{ij}\right)$, and molar volume of permeant (*v*_*i*_), consecutively. Whereas the operating condition that dominantly affects the PV permeation flux was permeate pressure (*P*_*p*_).

## Highlights

• PV flux is correlated to the properties of permeant-membrane and process conditions.
• The correlation is built by machine learning of artificial neural network (ANN).
• The output is PV flux, the inputs are material properties and process conditions.
• Several neural network architectures are evaluated.
• The dependency of output on input variables is determined with Garson's equation.

## Graphical abstract

**Figure:**
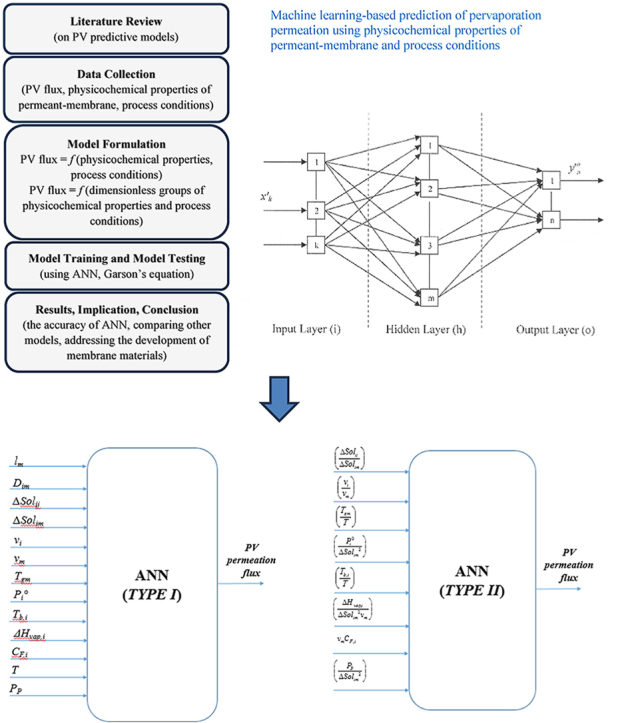

## 1 Introduction

Pervaporation (PV) is a membrane separation process extensively employed for the separation of liquid mixtures that are challenging to separate through conventional methods due to azeotropic behavior or close boiling points. The process utilizes a selective membrane to separate components based on their different chemical potential gradients, driven by partial vapor pressures across the membrane [1]. PV involves feeding a liquid mixture to one side of a dense, non-porous membrane, with the selective permeation of one or more components into the vapor phase on the opposite side, driven by a vacuum or a sweep gas. The performance of PV is commonly assessed using several metrics [2], i.e. permeation flux, selectivity or separation factor, and energy efficiency. The permeation flux indicates the capacity of membrane to permeate the favored component, which is generally expressed in term of mass flux. The selectivity or separation factor is a measure of the membrane's ability to preferentially separate one component over others. Since PV typically requires thermal energy for the feed and mechanical energy to maintain vacuum conditions, its energy efficiency is also a critical factor [2].

Despite its advantages, PV faces challenges that limit its wider industrial application. For example, accumulation of materials on the membrane surface can reduce its efficiency and lifespan. Moreover, high selectivity often comes at the cost of reduced permeability, necessitating the development of more advanced membrane materials. Additionally, long-term operation can lead to degradation of membrane properties due to chemical, thermal, and mechanical stresses [3]. As the PV permeation is basically affected by the physicochemical properties of the permeants-membrane materials used, many researchers developed membrane materials to improve their characteristics in permeating and selecting the permeant to be separated as well as resisting the membrane degradation. The development of membrane materials includes blended-polymeric membrane [[4], [5], [6], [7], [8]], crosslinked-polymeric membrane [[9], [10], [11]], grafted-polymeric membrane [[12], [13], [14], [15], [16]], polyelectrolyte complex membrane [[17], [18], [19], [20], [21]], and nano-composite membrane [[22], [23], [24]], from various sources of materials.

The development of membrane materials may be addressed through predictive models, which are derived from both the transport mechanism and the experimental data provided in any literature. For this purpose, understanding transport behavior of permeant in PV is mandatory. An accurate transport model is required so that the simulation of any PV process design can be close to real applications without conducting experimental works. In fact, several PV models have been developed over the years. All PV predictive models were derived from the concept of solution-diffusion model, which was applicable for non-porous membranes. However, most predictive models consider only one step of mass transfer of permeant to the membrane, either sorption or diffusion. The preferential sorption can be predicted from the polarity [25,26], interfacial thermodynamics [27,28], and chromatographic properties [[29], [30], [31], [32]]. Lipnizki et al. [33] categorized the developed models into three types, i.e. empirical, theoretical, and semi-empirical models. Each has advantages-disadvantages and ranges of applications. As no models are applicable satisfactorily for wide ranges of PV systems, Mujiburohman [34] proposed a hybrid model to predict the PV performance by taking into account all steps of transport mechanism in PV, i.e. sorption, diffusion, and desorption; while other previous studies ignored the desorption step. The permeation flux (*J*_*i*_) was defined as the multiplication between an overall permeation conductance (*k*_*overall,i*_) and a permeation driving force. The overall permeation conductance was correlated empirically to selected physicochemical properties of permeant-membrane and process conditions through dimensional analysis. The empirical constants were obtained from processing a large database of various PV systems using non-linear regression method and gave the average errors of 28.32 % and 30.65 % in the model training and its testing, respectively. It was also concluded that to some extent the desorption step could influence the permeation flux.

In most cases, a method so-called artificial neural network (ANN) gives better accuracy in the model training and testing in comparison with regression method. Elkamel et al. [35] applied ANN to predict ozone (O_3_) levels as a function of meteorological parameters (wind speed, wind direction, temperature, etc.) around heavily industrialized areas, and found that ANN model predictions gave average errors of 11.1 % and 12.5 % in the model training and testing, respectively, which were better than using linear and non-linear regression methods (average errors of 37.4 % and 12.84 %, respectively in the model training); while, in the testing the non-linear regression gave an average error of 20.04 %. Studies on using ANN as predictive models have also been conducted in the following research areas: glass transition temperature of multicomponent oxide glasses [36], microfiltration membrane fouling [37], ultrafiltration fouling [38], thermal behavior nanocomposite [39], removal of solid suspensions and chemical oxygen demand from a pharmaceutical wastewater plant [40], CO_2_ solubility in aqueous solutions of glycerol and monoethanolamine [41], phase equilibrium of ethanol-congener mixtures [42], biogas thermodynamic properties [43], wind power forecasting [44], hydrogenation degree prediction [45], hand gesture recognition for robotic arm control [46], classification of fMRI data [47], etc.; those studies found that ANN is successful in predicting the investigated systems. Briefly speaking, for complex processes where mechanistic models may be difficult to describe, ANN models are suitable to use as long as reliable and wide range of corresponding data is available for model training. Therefore, ANN modeling has been applied commercially in such processes including business applications (voice signal stabilization in communication system, risk analysis system, etc.), aerospace (aircraft autopilot, simulation of flight path, detection of aircraft component fault, etc.), automotive (automatic guidance system, analysis of warranty activity), banking (evaluation of credit application, mortgage screening, etc.), medical (cancer cell analysis, optimization of transplant times, etc.), and even chemical industries (process control, predicting output gases of furnace or other industrial processes, and oil-gas exploration).

Given some challenges encountered by PV and considering the accuracy of ANN for predictive models, there is a critical need to optimize PV processes to enhance their efficiency and viability supported by machine learning models. This study addresses these needs by employing ANN to predict and analyze the permeation flux of PV systems. By integrating machine learning with PV, this research aims to achieve the following detailed objectives.

1. To enhance the predictability of permeation flux based on physicochemical properties of the permeant-membrane system and operating conditions, thereby facilitating better design and operational strategies.
2. To identify critical factors influencing performance, thus enabling targeted improvements in membrane composition and process parameters.
3. To provide a deeper understanding of the dynamic interactions within the PV system, offering insights that are not readily apparent through traditional modeling approaches.

**Fig. 1:**
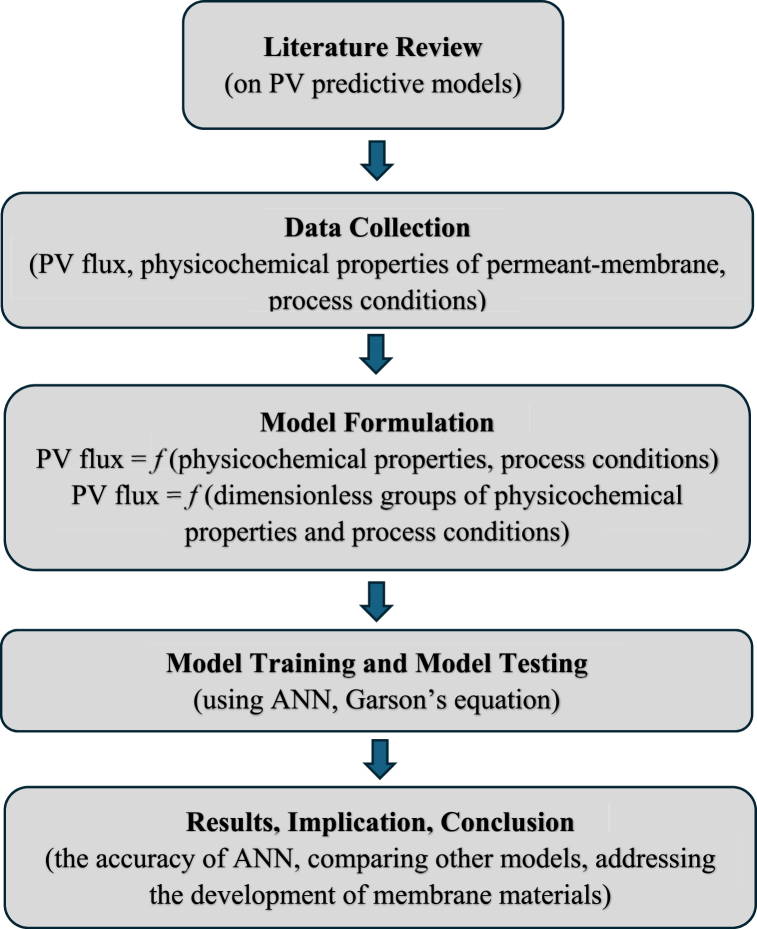
Scheme of study to construct ANN predictive models of PV permeation flux based on the physicochemical properties of permeants-membranes and process conditions.

By leveraging a dataset compiled from extensive literature and applying different neural network architectures, this study not only advances the fundamental understanding of PV dynamics but also enhances practical applications in industries where high-purity separation is crucial. Specifically, this study determines the architectures of ANN that give the best predictions both in the model training and its testing. The relative importance of each input on the output is also determined using Garson's equation [48]. Based on the input variables, two types of correlations of input-output are compared, i.e.: (i) Type I, the inputs consist of values of regular variables of physicochemical of permeant-membrane and process conditions; and (ii) Type II, the inputs are the dimensionless groups derived from the regular variables. The scheme of the study is briefly presented in Fig. 1.

## 2 Artificial neural network (ANN)

The concept of ANN imitates biological neural systems. In human beings any input of information caught by human senses is passed and processed by neural systems to result in an action output. Similar data will be treated in the same way and result in similar output. At this point, the neural systems identify certain input with certain pattern of treatment to give certain output. Similarly, ANN models comprise large connections between elements so-called neurons. These neurons consist of specific functions to relate the output or target values with the given input. The correlation between output and input is trained by ANN until the required minimum error (difference between ANN predictions and real data) of target values is met by adjusting the values of connection parameters so-called weights (*w*).

**Fig. 2:**
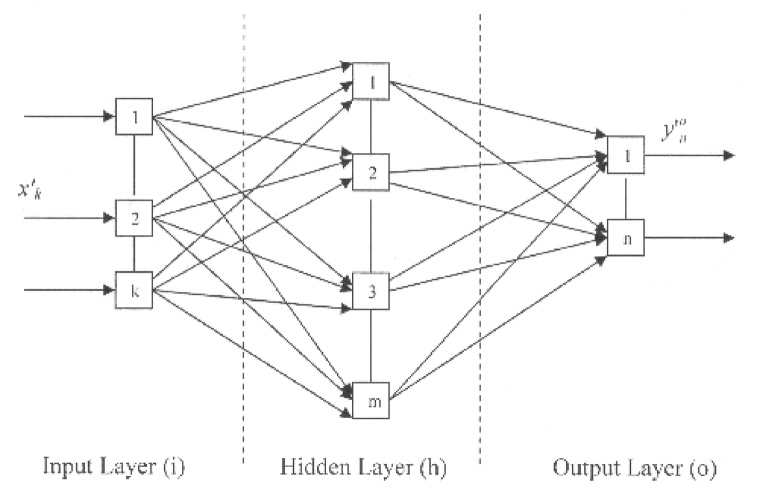
Typical architecture of ANN.

An ANN architecture is composed of at least three layers of neurons, as follows: one layer of input neurons (i), one layer of hidden neurons (h), and one layer of output neurons (o). An example of ANN architecture with one hidden layer is described in Fig. 2 [35].

The input neurons act as distribution channels that transmit the initial input into hidden neurons. No transfer functions take place in the input layer, and thus the output of the input layer is the same as its input [35].

(1) $$y_{k}^{\prime \prime }=x_{k}^{\prime }$$

*x'* and *y”* represent the normalized values of initial (raw) input and calculated output which are defined as follows [35],

(2) $$x^{\prime }=\frac{x-x_{\min}}{x_{\max}-x_{\min}}$$

where *x*, *x*_min_, and *x*_max_ are the actual value of raw input, the minimum value of raw input, and the maximum value of raw input, respectively. The values of variables are normalized in order that they have the same range of values with the boundaries of transfer function used in the hidden layer. Before being transferred, the output from the input layer is processed by hidden neurons using weighted summation, as follows [35]:

(3) $$w_{km}^{ih}x_{m}^{h}=\sum_{k=0}^{k}y_{k}^{i}w_{km}^{ih}+b_{m}$$

$x_{m}^{h}$ represents the weight factor emanating from neuron *k* in input layer *i* and terminating at neuron *m* in hidden layer *h*. *b*_*m*_ is the value of “bias” factor associated to neuron *m*. Bias neurons are added in the hidden and output layers to provide universal approximation of ANNs. Subsequently, becomes new input for hidden layer and then is transferred by hidden neurons into certain output. One of the most widely used transfer function is the ‘S’-shaped logistic sigmoid (logsig) [35]:

(4) $$f\left(x\right)=\frac{1}{1+\exp\left(-x\right)}$$

$x_{m}^{h}y_{m}^{h}$ This transfer function has certain unique features such as continuously differentiable, monotonic, symmetric, and bounded between 0 and 1. Inputting into Eqn. (4) results in output of hidden layer (.) [35]:

(5) $$y_{m}^{h}=\frac{1}{1+\exp\left(-x_{m}^{h}\right)}$$

Since there is no transfer function in the output layer, the output of the output layer is the same as its input. However, like the input of hidden layer, the input of output layer comes from the output of previous layer (i.e. hidden layer) that has been treated with weighted summation, as follows [35]:

(6) $$x_{n}^{o}=\sum_{m=0}^{m}y_{m}^{h}w_{mn}^{ho}+b_{n}$$

Then, the output of output layer will be [35]:

(7) $$y_{n}^{o}=x_{n}^{o}$$

ANN train the model until the required minimum error (*E*_*j*_) is achieved. *E*_*j*_ is defined as [35]:

(8) $$E_{j}=\sum_{j=1}^{n}{\left(y_{nj}^{\prime }-y_{nj}^{o}\right)}^{2}$$

It can be stated that model training in ANN is essentially an optimization process to minimize error function as given in Eqn. (8) by adjusting the weights *w* in the hidden layer.

## 3 Data collection

The dataset for model training and testing was adopted from Ref. [34] which was originally collected from other PV studies published in various journals [[49], [50], [51], [52], [53], [54], [55], [56], [57], [58], [59], [60], [61], [62], [63], [64]]. There were 144 points in total; roughly 90 % of it (131 points) was used for model training, and the remaining points were used for model testing. Not all experimental data of PV published in any journals were used in this study because the data must consist of all the variables required in the proposed types of correlations including: permeation flux (*J*_*i*_), permeant feed concentration (*C*_*F,i*_), operating temperature (*T*), permeate pressure (*P*_*P*_), membrane thickness (*l*_*m*_), diffusivity of permeant through membrane (*D*_*im*_), the molecular interactions of permeant-permeant and permeant-membrane which are represented by their solubility parameter differences (*ΔSol*_*ij*_, *ΔSol*_*im*_), molar volume of permeant (*v*_*i*_), molar volume of polymeric membrane (*v*_*m*_), glass transition temperature of polymeric membrane (*T*_*gm*_), vapor pressure of permeant at reference temperature (*P*_*i*_^*o*^), boiling point of permeant at reference pressure (*T*_*b,i*_), and vaporization heat of permeant at reference temperature and pressure (*ΔH*_*vap,i*_). The solubility differences of permeant-permeant and permeant-membrane are defined as [34],

(9) $$\Delta Sol_{ij}=\Delta Sol_{ji}=\left|\delta_{j}-\delta_{i}\right|$$

(10) $$\Delta Sol_{im}=\left|\delta_{m}-\delta_{i}\right|$$

where *δ*_*i*_, *δ*_*j*_, and *δ*_*m*_ are the solubility parameters of permeant *i*, permeant *j*, and membrane *m*, respectively. The *D*_*im*_ used in this work was not intrinsic diffusivity but was calculated using LaPack correlation which was a permeant molar volume (*v*_*i*_) dependence [65].

(11) $$D_{im}=8.25x10^{-4}\;\exp\left(-1.06v_{i}^{1/3}\right)$$

## 4 Neural network model training

Two types of correlations of input-output are compared in this work. Type I is the correlation between permeation flux (*J*_*i*_) versus each single variable considered to affect the PV permeation flux, as follows [34],

(12) $$J_{i}=f\left(l_{m},D_{im},\Delta Sol_{ij},\Delta Sol_{im},v_{i},v_{m},T_{gm},P_{i}^{o},T_{b,i},\Delta H_{vap,i},C_{F,i},T,P_{P}\right)$$

Type II utilizes a scaled version (dimensionless group) correlated directly to the permeation flux *J*_*i*,_ instead of to the overall permeation conductance (*k*_*overall*_) as proposed in Ref. [34], as follows:

(13) $$J_{i}=f\left(\left(\frac{\Delta Sol_{ij}}{\Delta Sol_{im}}\right),\left(\frac{v_{i}}{v_{m}}\right),\left(\frac{T_{gm}}{T}\right),\left(\frac{P_{i}^{o}}{\Delta Sol_{im}^{2}}\right),\left(\frac{T_{b,i}}{T}\right),\left(\frac{\Delta H_{vap,i}}{\Delta Sol_{im}^{2}v_{m}}\right),\left(v_{m}C_{F,i}\right),\left(\frac{P_{P}}{\Delta Sol_{im}^{2}}\right)\right)$$

**Fig. 3:**
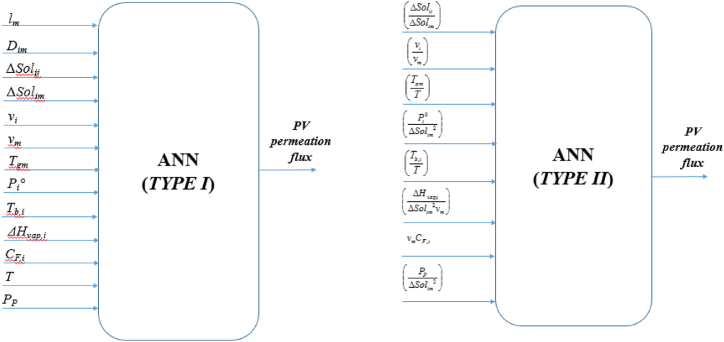
Model structure for ANN Type I and ANN Type II.

It should be clarified that for ANN Type I, it is commonly found in the ANN modeling that the input variables are any individual physical quantities which are considered to affect the output variable, i.e. PV flux. On the other hand, Type II is intended to reduce the number of input variables by converting the individual variables into dimensionless groups through dimensional analysis as obtained in Ref. [34]. The difference in model structure from input-output perspective for the 2 types of NN models is demonstrated in Fig. 3.

The ranges of values (i.e., minimum and maximum values) of variables in Eqn. (12) and dimensionless groups in Eqn. (13) are given in Table 1, Table 2, respectively.

**Table 1:** The minimum and maximum values of regular variables.

No.	Variable (Dimension)	Symbol	Minimum Value	Maximum Value
1	Thickness of membrane, (length)	*l*_*m*_	0.000003	0.000163
2	Diffusivity of permeant through membrane, (length^2^/time)	*D*_*im*_	3.729 × 10^−6^	5.102 × 10^−5^
3	Solubility parameter difference between permeant i and j, (energy/volume)^1/2^	*ΔSol*_*ij*_	21.3	30.2
4	Solubility parameter difference between permeant i and membrane m, (energy/volume)^1/2^	*ΔSol*_*im*_	1.0	32.9
5	Molar volume of permeant, (volume/mass)	*v*_*i*_	18.1	132.15
6	Molar volume of membrane, (volume/mass)	*v*_*m*_	36.8	172.0
7	Glass-transition temperature of polymeric membrane, (temperature)	*T*_*gm*_	150.15	259.5
8	Saturated vapor pressure of permeant, (pressure)	*P*_*i*_*°*	0.000833	0.306491
9	Boiling point of permeant, (temperature)	*T*_*b,i*_	329.35	457.45
10	Heat of vaporization of permeant, (energy/mass)	*ΔH*_*vap,i*_	23698.18	43124.49
11	Concentration of permeant *i* in the feed bulk, (mass/volume)	*C*_*F,i*_	8.383	997990.4
12	Operating temperature, (temperature)	*T*	296.15	353
13	Permeate pressure, (pressure)	*P*_*P*_	0.000167	0.029998

**Table 2:** The minimum and maximum values of dimensionless groups.

No.	Dimensionless Group	Minimum Value	Maximum Value
1	$\left(\frac{\Delta Sol_{ij}}{\Delta Sol_{im}}\right)$	0.9027	29.6
2	$\left(\frac{v_{i}}{v_{m}}\right)$	0.1052	3.1136
3	$\left(\frac{T_{gm}}{T}\right)$	0.4953	0.8704
4	$\left(\frac{P_{i}^{0}}{\Delta So{l_{im}}^{2}}\right)$	2.1904 × 10^−5^	4.6652 × 10^−2^
5	$\left(\frac{T_{b,i}}{T}\right)$	1.0571	1.5158
6	$\left(\frac{\Delta H_{vap,i}}{\Delta So{l_{im}}^{2}v_{m}}\right)$	37.5876	33200.04
7	$v_{m}C_{F,i}$	1441.9085	171654348.8
8	$\left(\frac{P_{P}}{\Delta So{l_{im}}^{2}}\right)$	1.068 × 10^−6^	3.9444 × 10^−3^

Training the dataset using Matlab finds the optimum architecture of ANN to use. The comparison of ANNs architectures together with precisions for both types of correlations is given in Table 3, Table 4.

**Table 3:** Various ANN architectures and performance for Type I data set.

Parameter	Shallow NN						
Number of hidden layers	1	1	1	1	1	1	1
Number of neurons	6	7	8	9	10	11	12
Performance							
- Training: *R*	0.88135	0.95834	0.92299	0.88413	0.85814	0.94124	0.88774
-Validation: *R*	0.72648	0.73144	0.85373	0.80995	0.60276	0.62865	0.62631
-All: *R*	0.85066	0.87298	0.90715	0.87129	0.81981	0.87885	0.81551
-Testing: RMSE	42.5239	101.1288	76.7761	47.2192	56.0875	59.3312	49.474

Parameter	Deep NN 2 Layers			
Number of hidden layers	2	2	2	2
Number of neurons	5 x 5	7 x 7	10 x 10	11 x 11
Performance				
-Training: *R*	0.66756	0.93674	0.26485	0.94262
-Validation: *R*	0.58269	0.93091	0.50747	0.62311
-All: *R*	0.64277	0.93036	0.33003	0.90758
-Testing: RMSE	56.766	42.2558	111.3595	66.3574

Parameter	Deep NN 3 Hidden Layers		
Number of hidden layers	3	3	3
Number of neurons	5 x 5 x 5	7 x 7 x 7	10 x 10 x 10
Performance			
-Training: *R*	0.67627	0.96314	0.091696
-Validation: *R*	0.1487	0.71283	0.25125
-All: *R*	0.60644	0.93324	0.1293
-Testing: RMSE	33.6935	50.5883	100.6553

**Table 4:** Various ANN architectures and performance for Type II data set.

Parameter	Shallow NN			
Number of hidden layers	1	1	1	1
Number of neurons	6	7	8	9
Performance				
-Training: *R*	0.88332	0.82222	0.76682	0.82432
-Validation: *R*	0.76572	0.46392	0.87426	0.94712
-All: *R*	0.85564	0.73144	0.78112	0.78112
-Testing: RMSE	38.0766	52.9532	41.1479	40.6118
Parameter	Deep NN 2 Layers			

Number of hidden layers	2	2	2	2
Number of neurons	5 x 5	7 x 7	9 x 9	11 x 11
Performance				
-Training: *R*	0.76716	0.629	0.71439	0.83017
-Validation: *R*	0.83931	0.56885	0.78911	0.50735
-All: *R*	0.78285	0.61137	0.74089	0.75792
-Testing: RMSE	50.1307	49.5172	48.5776	44.5235

Parameter	Deep NN 3 Hidden Layers		
Number of hidden layers	3	3	3
Number of neurons	5 x 5 x 5	7 x 7 x 7	9 x 9 x 9
Performance			
-Training: *R*	0.85161	0.56334	0.88789
-Validation: *R*	0.7918	0.49584	0.66529
-All: *R*	0.83979	0.56402	0.84229
-Testing: RMSE	49.9303	56.288	41.3956

**Fig. 4:**
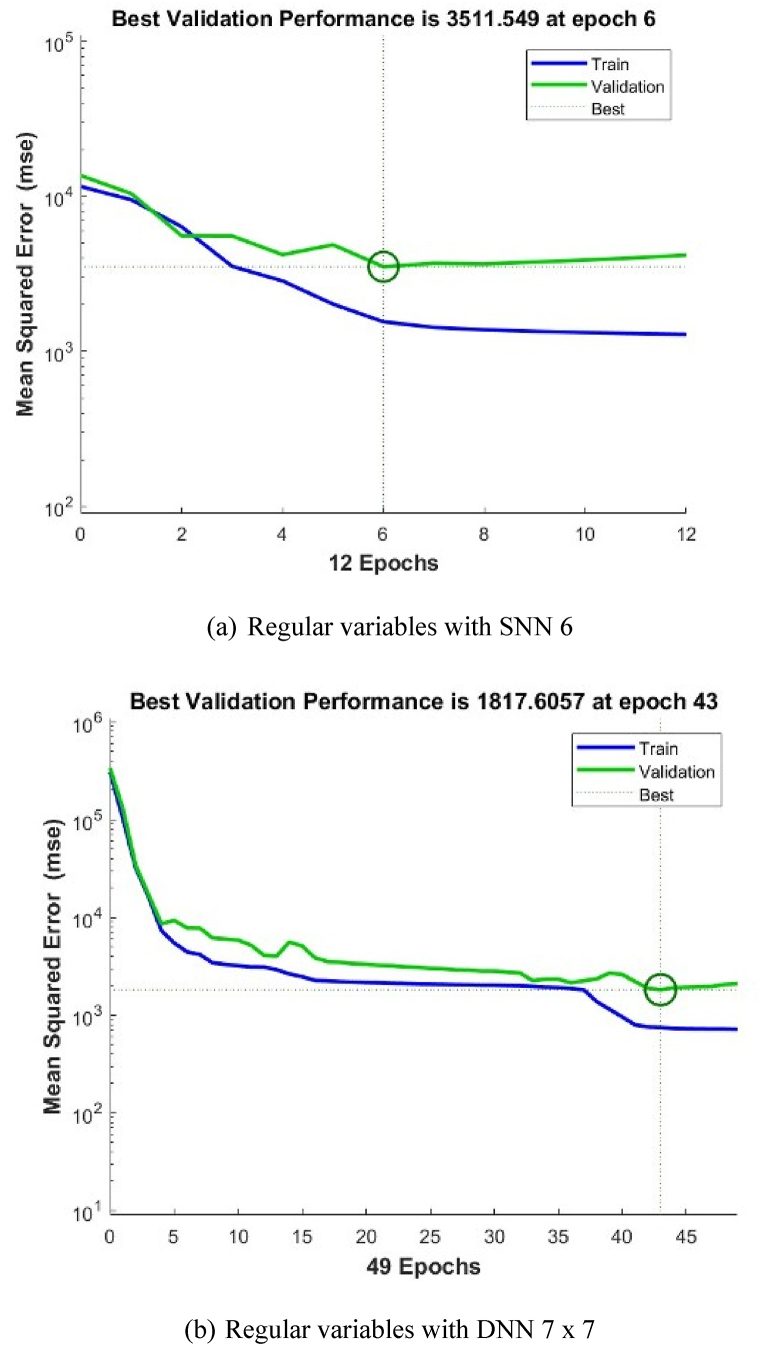
Validation performance of PV permeation flux vs regular variables (Type I).

**Fig. 5:**
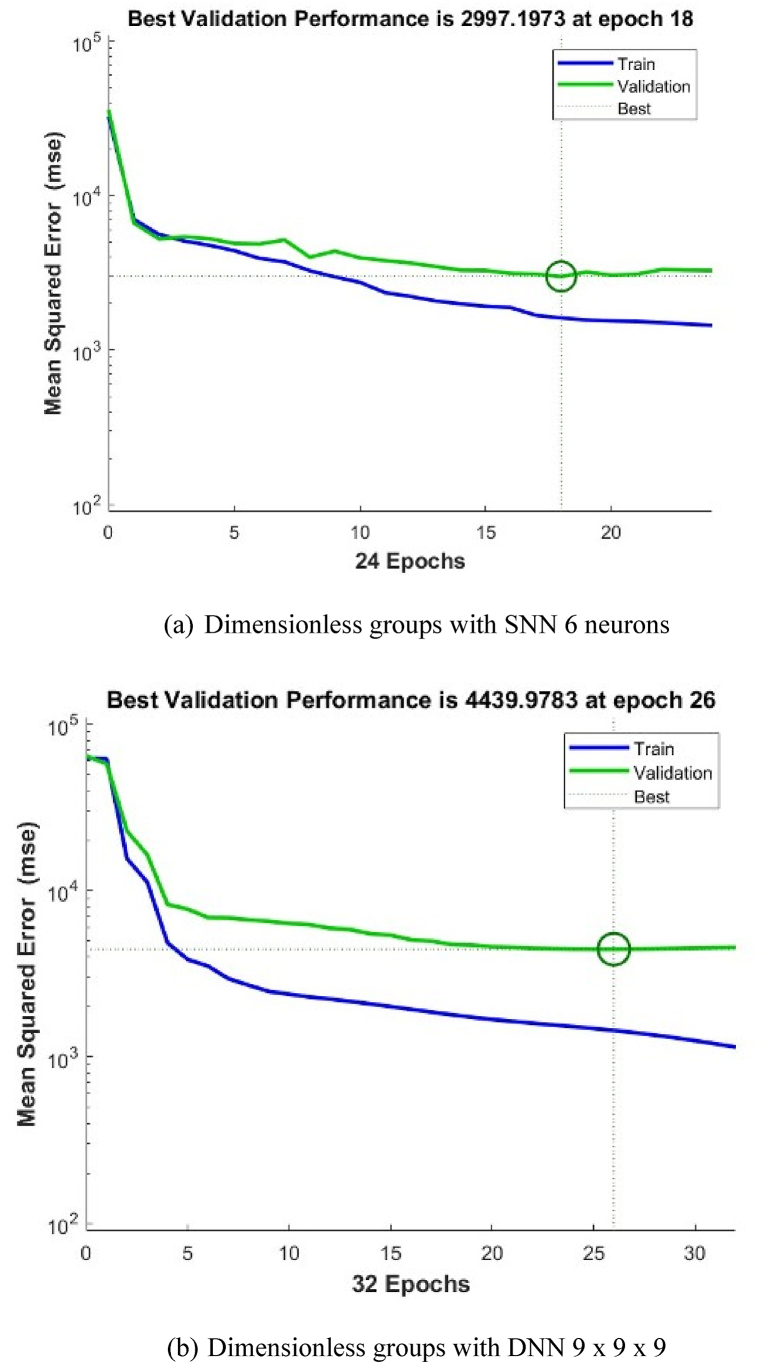
Validation performance of PV permeation flux vs dimensionless groups (Type II).

**Fig. 6:**
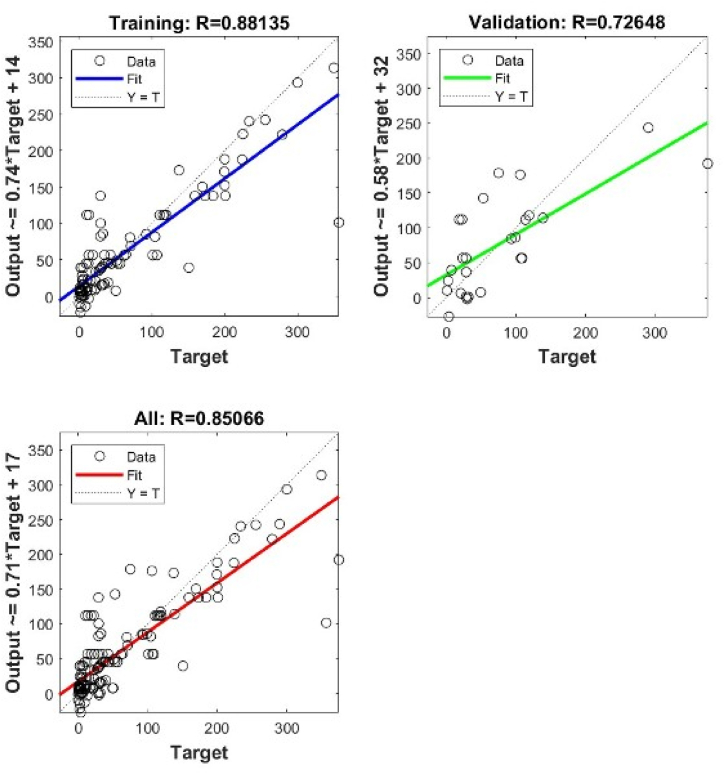
Cross plots and *R* values of SNN 6 neurons for Type I.

**Fig. 7:**
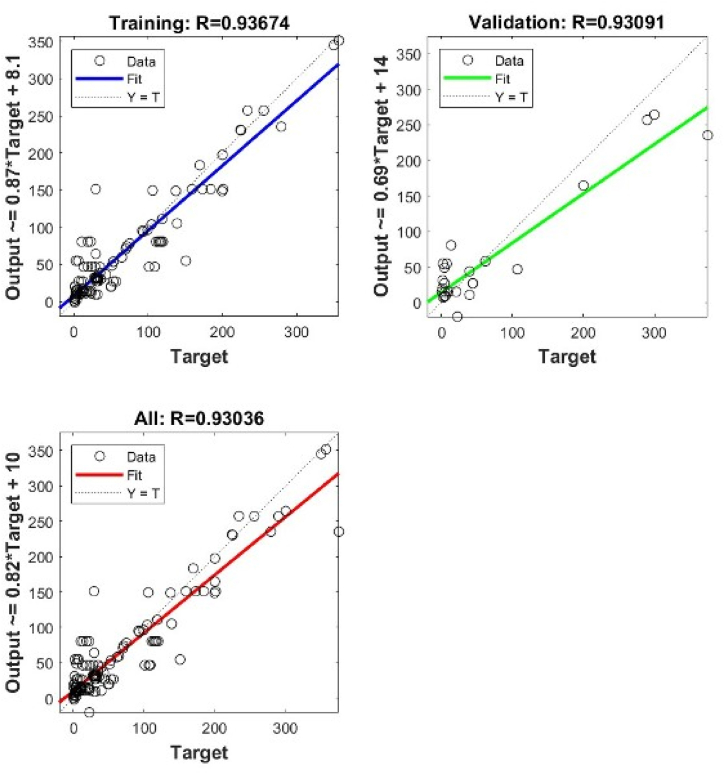
Cross plots and *R* values of DNN 7 x 7 neurons for Type I.

**Fig. 8:**
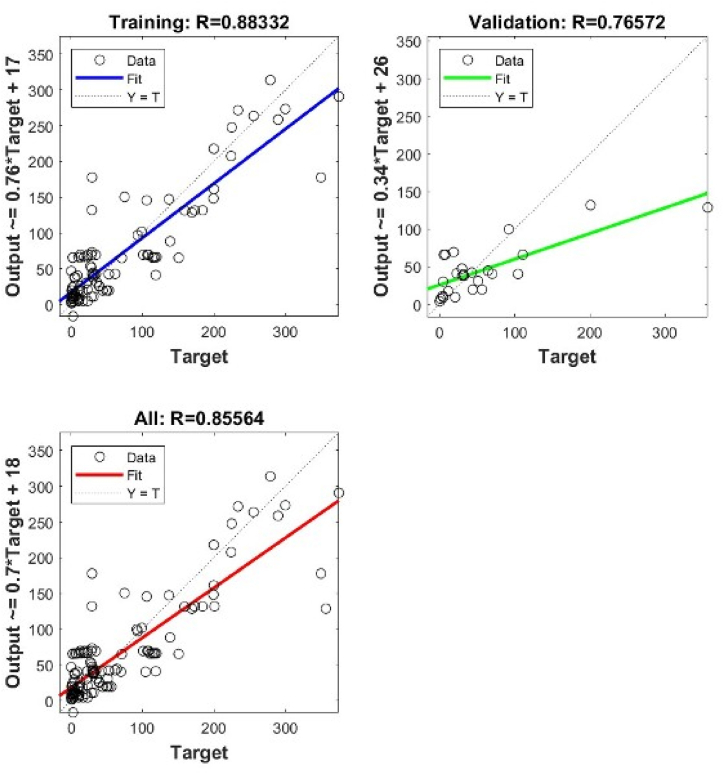
Cross plots and *R* values of SNN 6 neurons for Type II.

**Fig. 9:**
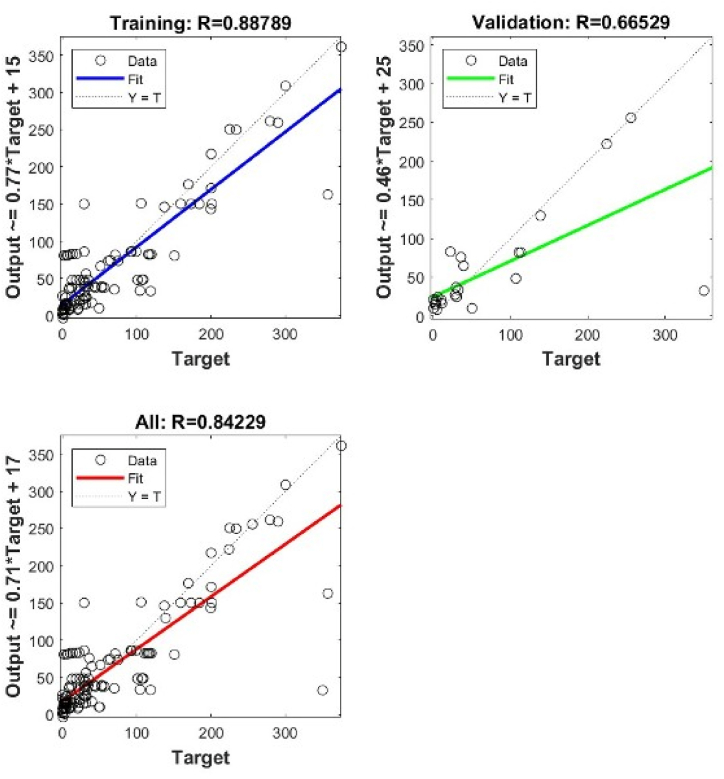
Cross plots and *R* values of DNN 9 x 9 x 9 neurons for Type II.

In general, the shallow NN with a single layer of 6 neurons gives the best precisions for both types. However, when using deep NN with more hidden layers and different architectures, the best precisions are obtained when we have 2 layers 7 x 7 for Type I, and 3 layers 9 x 9 x 9 for Type II. The validation performance of these architectures is shown in Fig. 4, Fig. 5, Fig. 6, Fig. 7, Fig. 8, Fig. 9.

Using a single hidden layer, Type II gives better precisions than Type I. The reason may relate to the number of input nodes. Converting the regular variables into dimensionless groups reduces the number of input nodes, from 13 into 8. To further verify the validity of model training, the weights obtained in the model training are used to predict the other 13 points of experimental *J*_*i*_.

## 5 Neural network model testing

**Fig. 10:**
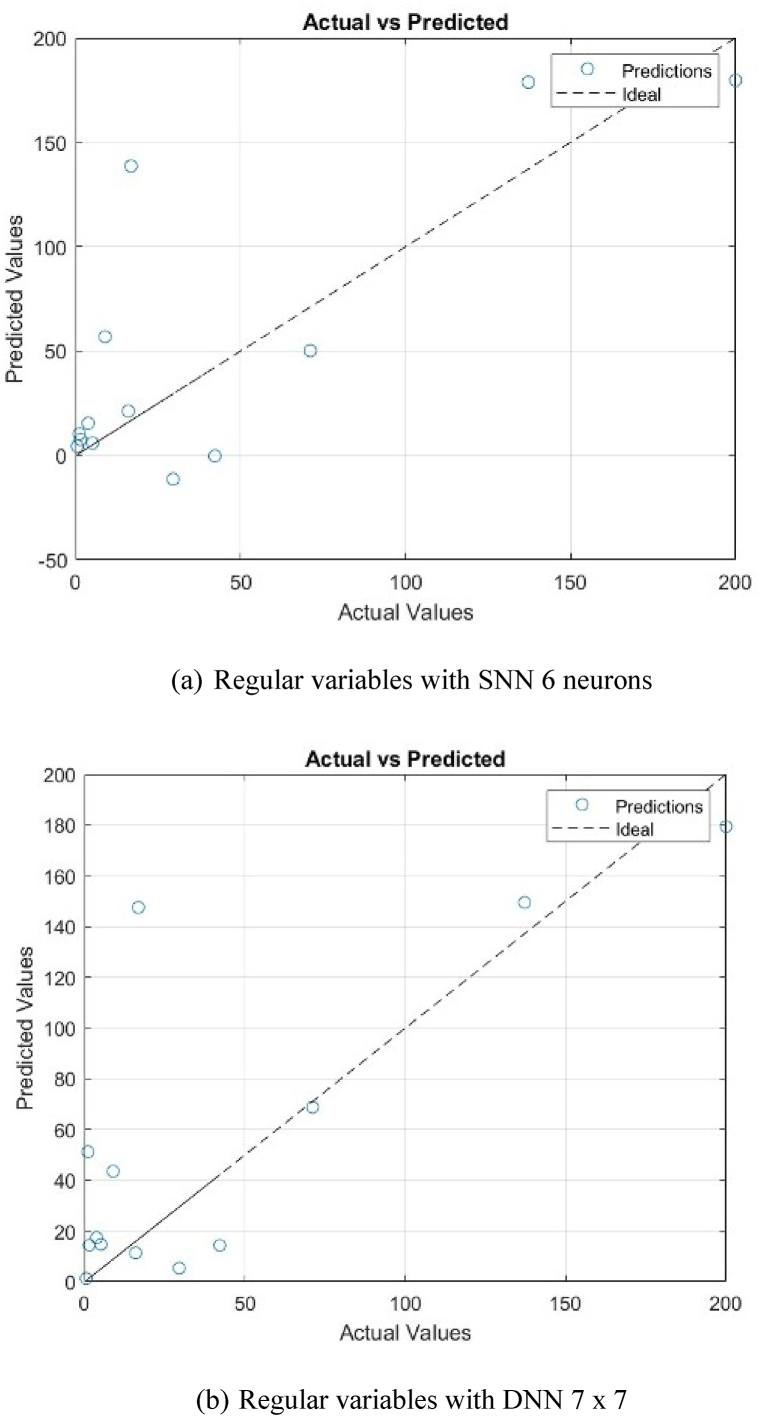
A cross plot comparing the experimental *J*_*i*_ and the predicted *J*_*i*_ for Type I.

**Fig. 11:**
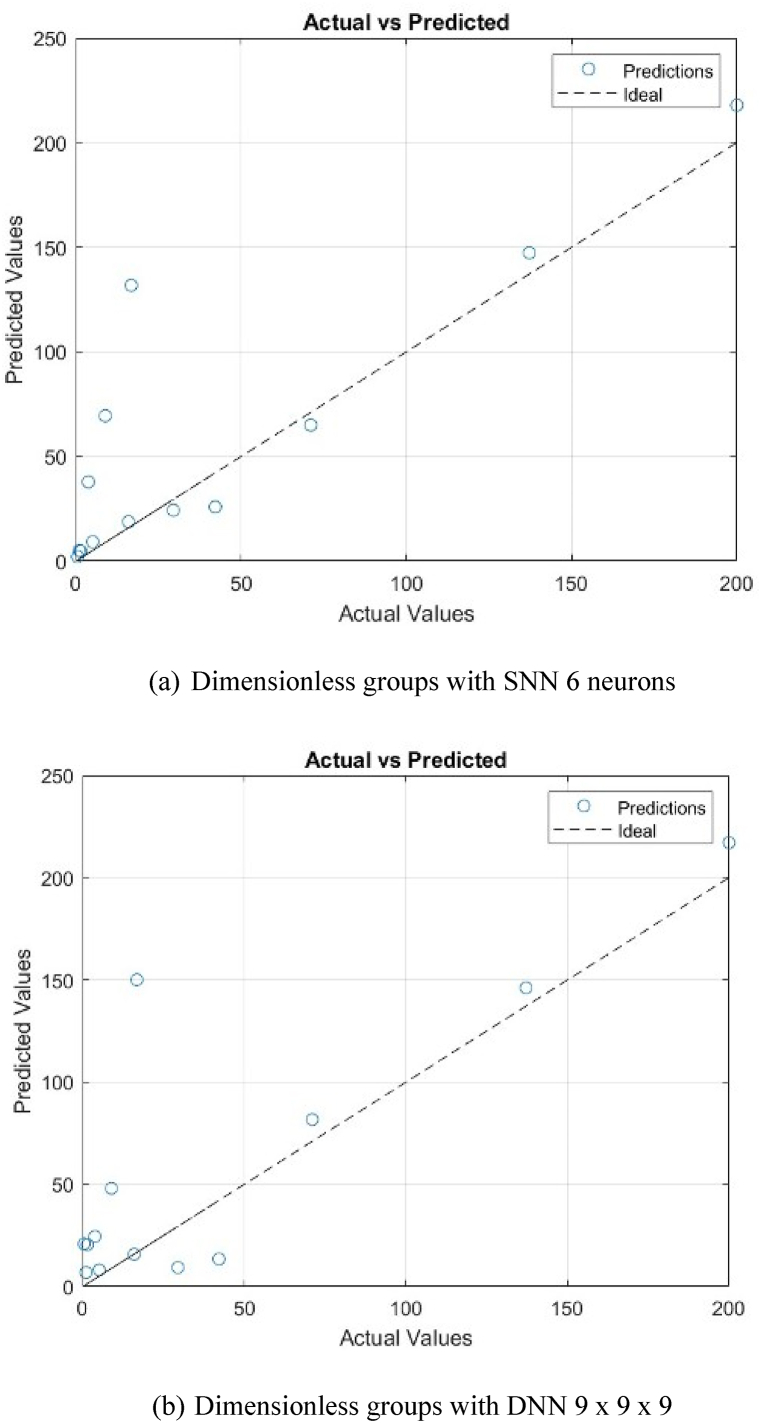
A cross plot comparing the experimental *J*_*i*_ and the predicted *J*_*i*_ for Type II.

Using the weights obtained in the model training, the ANN models for both types of correlations give good predictions on the permeation fluxes of the tested PV systems. As in the model training, Type II also gives better precisions with RMSE of 38.0766; while the RMSE of Type I is 42.5239. A cross plot comparing the experimental *J*_*i*_ and the predicted *J*_*i*_ for both types of correlations is shown in Fig. 10, Fig. 11.

It can be seen that the experimental and the predicted data points are relatively at 1:1 parity line, indicating that there is a good agreement between model predictions and experimental data.

In comparison with the non-linear regression method as proposed in Ref. [34], for similar correlation of input-output (Type II), the non-linear regression method gives a better *R*^*2*^ (0.9721616). It may be stated that a model derived from more detailed process mechanism of the system gives better correlation [34]. correlated the dimensionless groups of physical properties of permeant-membrane and process conditions, to a quantitative parameter so-called permeation conductance (*k*_*overall,i*_). The permeation conductance was then multiplied with the driving force of permeation, which was the permeant concentration difference between both sides feed and permeate, and was further simplified to be the concentration of permeant in the feed solution (*C*_*F,i*_). Whereas, in this study the dimensionless groups of physical properties of permeant-membrane and process conditions are correlated directly to the permeation flux.

It must be noted that in case the correlation of input-output is not built based on process mechanism like Type I, ANNs model may fail as extrapolator, where the input values for predicting the tested systems are out of the ranges of input values in the model training. Thus, it is suggested that in order that ANNs model acts as a good extrapolator and can be applied for wider PV systems, the correlation of input-output must be developed by considering the mechanism of the process.

## 6 Extent of variable dependency

The extent of dependency of output variable on each input variable can be determined based on partitioning of the connection weights of each hidden neuron into components associated with each input neuron. Garson [48] has provided a correlation for this purpose, as follows,

(14) $$IM\left(X_{p}\right)=\frac{\sum_{j=1}^{N_{p}}\left[\left(\frac{{\left|I\right|}_{pj}}{\sum_{k=1}^{N_{p}}{\left|I\right|}_{pj,k}}\right){\left|O\right|}_{j}\right]}{\sum_{i=1}^{N_{p}}\left\{\sum_{j=1}^{N_{p}}\left[\left(\frac{{\left|I\right|}_{pi,j}}{\sum_{k=1}^{N_{p}}{\left|I\right|}_{pi,j,k}}\right){\left|O\right|}_{j}\right]\right\}}$$

**Fig. 12:**
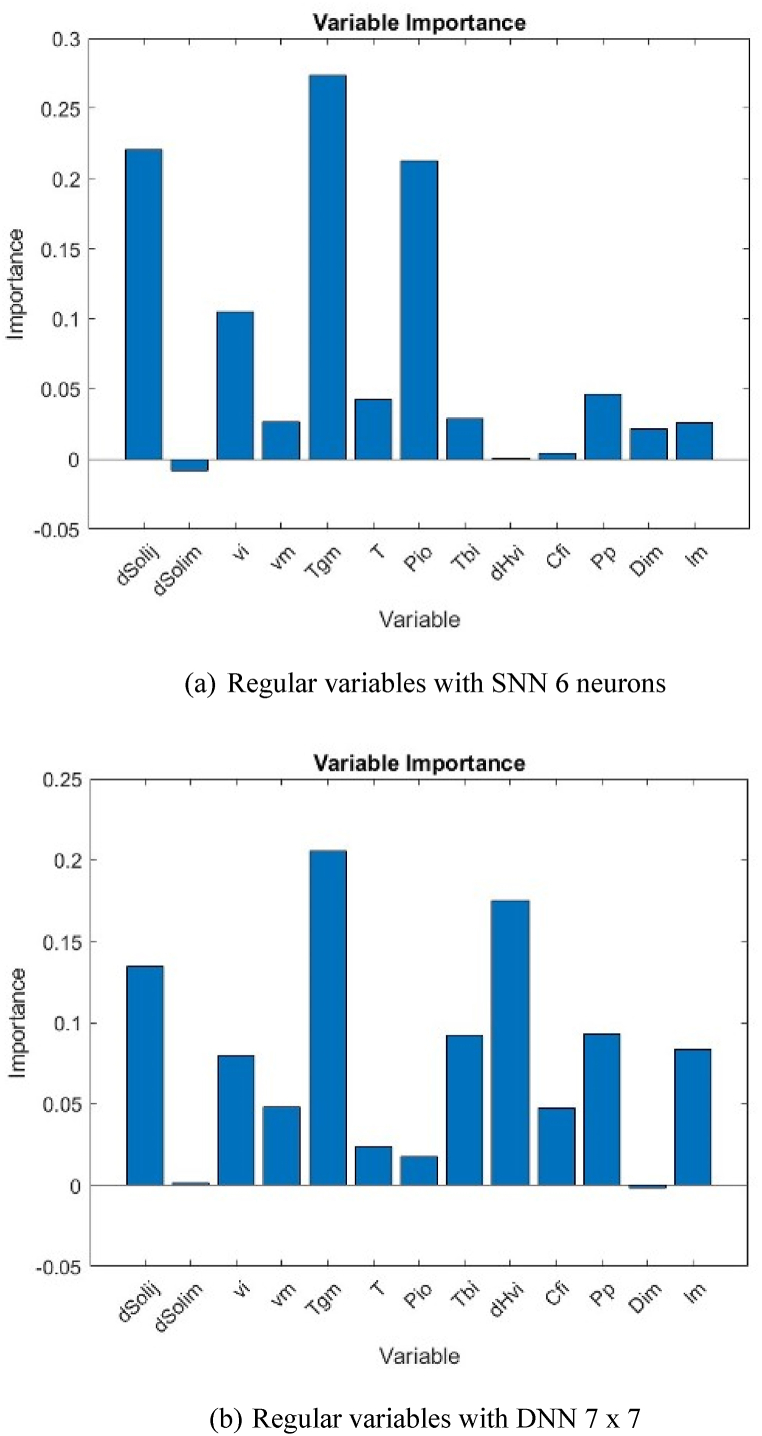
Variable importance of Type I.

**Fig. 13:**
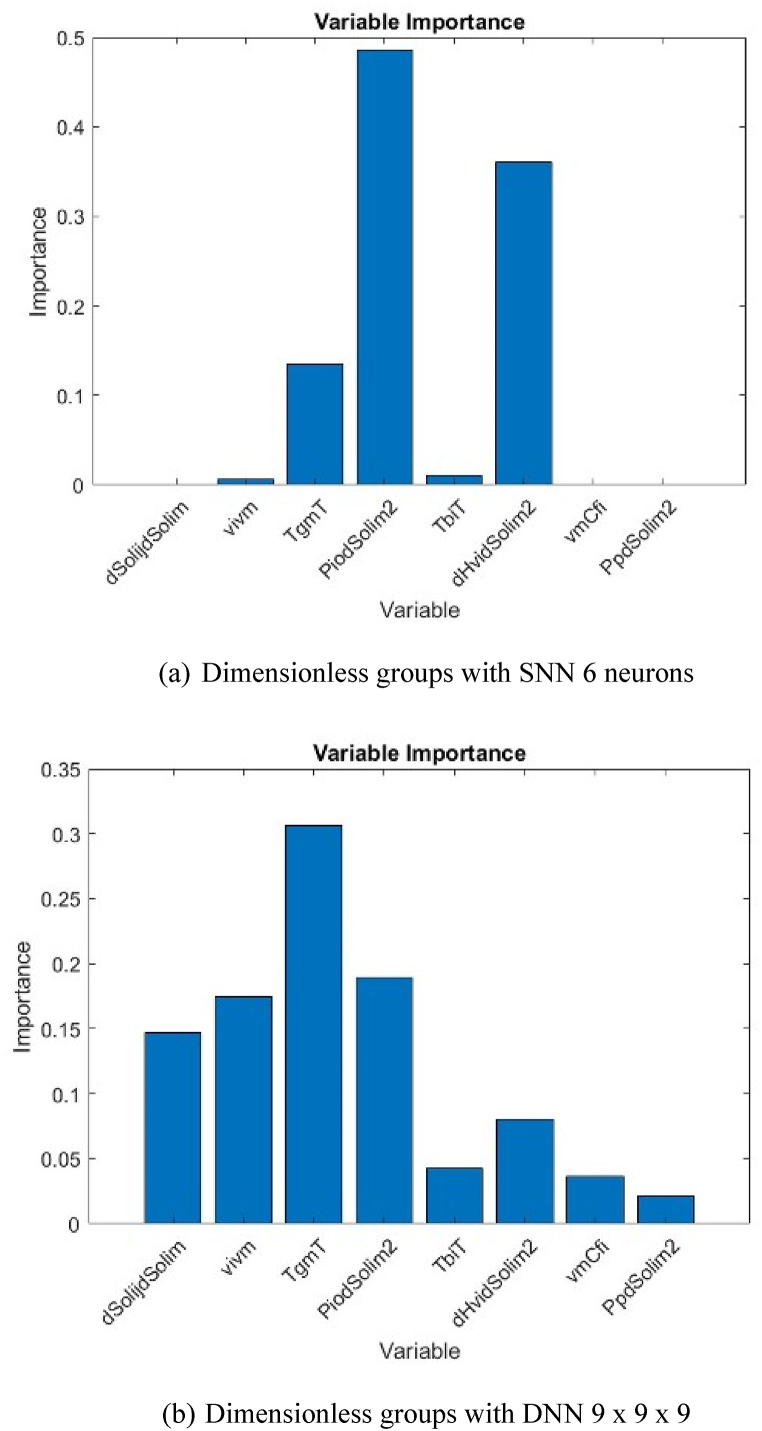
Variable importance of Type II.

${\left|O\right|}_{j}{\left|I\right|}_{pj}$ where *IM*(*X*_*p*_) is the importance measure for *p*th input variable *X*_*p*_. is the absolute value of the weight corresponding to the *p*th input variable and the *j*th hidden layer. is the absolute value of the weight in the output layer corresponding to the *j*th hidden layer. *N*_*p*_ is the number of input variables, which is in our case 13 for Type I and 8 for Type II. Using Eqn. (14), the extent of dependency of output to input variables in each type is shown in Fig. 12, Fig. 13, respectively.

For Type I, the most important physicochemical property and process condition that affect the PV permeation flux are the glass-transition temperature (*T*_*gm*_) and the permeate pressure (*P*_*p*_), respectively. All the data used in the model training and testing apply membranes made of polymeric materials. Polymers have temperature boundary at which there are significant changes of properties of polymers below and above the boundary; it is called glass-transition temperature [66]. Under the glass-transition temperature, the polymeric membrane is in the glassy state, and the permeant is difficult to pass through the membrane. It is vice versa when the polymeric membrane is above the glass-transition temperature, the polymeric membrane becomes rubbery, and the permeant can diffuse more easily. Thus, the two different states significantly change the PV permeation flux.

The effect of permeate pressure on the PV permeation flux is also significant when an atmospheric and vacuum conditions are compared. If the permeate pressure is atmospheric, the difference in chemical potential of permeant between both sides feed and permeate membrane surfaces, another term of PV driving force, is small thus low permeation flux. This is vice versa when the permeate pressure is high vacuum, the driving force of PV becomes high and thus high permeation flux.

As shown in Fig. 12, there are three physical properties that consistently give a significant affect to the PV permeation flux, regardless the types of neural networks (NN), i.e. glass-transition temperature (*T*_*gm*_), solubility difference between two different permeants (*ΔSol*_*ij*_), and molar volume of permeant (*v*_*i*_). It is well accepted that the molecular interactions of permeant-permeant affect the transport behavior of permeant through the membrane, particularly in the sorption step. These molecular interactions are proportional to the solubility difference in which the higher solubility difference between the two permeants will ease the sorptive separation [67]. The permeant having closer solubility to the membrane is preferentially sorbed on the membrane surface and diffuses through the membrane due to concentration gradient. The diffusion of permeant through membrane is affected by the molecular size of permeant, which is proportional to its molar volume [65]. The smaller molar volume of permeant will diffuse more easily, and thus a higher permeation flux can be achieved. Among those above physicochemical properties, the glass-transition temperature and the solubility difference relate directly to characteristics of membrane materials. Therefore, the development of membrane materials should be addressed to the synthesis of membrane materials having a low glass-transition temperature and a close solubility difference corresponding to the targeted permeant.

For Type II, when a DNN structure is used, a combination of glass-transition temperature and operating temperature (*T*_*gm*_/*T*) is found to be the most important dimensionless group affecting the PV permeation flux. Interestingly, when using SNN, a combination of saturated vapor pressure and solubility difference between permeant-membrane $\left(\frac{P_{i}^{0}}{\Delta So{l_{im}}^{2}}\right)$ becomes the most affecting physicochemical property to affect the PV permeation flux. The variable importance shows a good agreement with the mass transport mechanism of permeant through membrane widely accepted in PV, i.e. sorption, diffusion, and desorption. The sorption step is affected by the solubility difference between the two permeants (*ΔSol*_*ij*_) in which higher solubility difference will be easier to separately sorbed onto the membrane surface. The diffusion step is dominantly affected by the molecular size of permeant (represented by *v*_*i*_); whereas the desorption step is usually ignored. However, the saturated vapor pressure (*P*_*i*_^*0*^), which is proportional to the ability of permeant to evaporate, was found to affect the PV permeation flux significantly. The variable importance of $\left(\frac{P_{i}^{0}}{\Delta So{l_{im}}^{2}}\right)$ is the highest among others, almost 0.5. This fact reveals that desorption step where the evaporation of permeant takes place needs to be considered, especially when the permeate pressure is low vacuum.

It is well understood that the accuracy of predictive models by machine learning is influenced by several factors including the validity of dataset used, the appropriate method of data processing, and the properness in selecting the subset of data in the model training and model testing. The larger number of valid data used is better in which the weigh factors obtained in the model training are applicable for wider range of input values in the model testing. Apart from ANN, several machine learning methods can also be used to process dataset to construct predictive models, such as multiple regression, random forest, support vector, bagging tree, and regression tree. Lastly, the selection of subset data of training and testing is also crucial. This is due to the fact that machine learning models are good as an interpolator, but often fail as an extrapolator. All these issues are potentially considered in the future work.

## 7 Conclusions

The use of ANN model for predicting the permeation flux in PV as a function of physicochemical properties of permeant-membrane and process conditions was investigated in this work. The output variable is the PV permeation flux; whereas the input variables are the physicochemical properties of permeant-membrane and process conditions which are considered to affect the PV permeation flux. Two types of inputs, i.e. regular variables (Type I) and dimensionless groups derived from regular variables (Type II) were trained with single neural networks (SNN) and deep neural networks (DNN). SNN with 6 neurons gives the best accuracy for both Types I and II; however, by adding number of hidden layers, better accuracy is found, i.e. DNN with 2 hidden layers of 7 x 7 neurons for Type I, and DNN with 3 hidden layers of 9 x 9 x 9 neurons for Type II. ANN model is proven to be able to predict the PV permeation flux based on the physicochemical properties of permeant-membrane and process conditions. ANNs model can also identify the relative importance of each input variable on the output variable. The glass-transition temperature (*T*_*gm*_), solubility difference between two different permeants (*ΔSol*_*ij*_), and molar volume of permeant (*v*_*i*_) are found the most affecting physicochemical properties to affect the PV permeation flux. The operating condition that dominantly affects the PV permeation flux is permeate pressure (*P*_*p*_).

To recap, this research has successfully met its objectives by leveraging advanced ANN methodologies to enhance the predictability and understanding of PV dynamics. The integration of machine learning with PV has facilitated more effective design and operational strategies and pinpointed the critical factors for optimizing membrane composition and process parameters. Overall, the study significantly contributes to the body of knowledge in industrial applications where the precision of high-purity separations is paramount, potentially leading to broader implementation and technological advancements in the field.

## CRediT authorship contribution statement

**Muhammad Mujiburohman:** Writing – original draft, Methodology, Conceptualization. **Marwen Elkamel:** Writing – review & editing, Validation, Software, Resources. **Farzad Hourfar:** Writing – review & editing, Validation. **Ali Elkamel:** Writing – review & editing, Validation, Software, Resources.

## Declaration of competing interest

The authors declare that they have no known competing financial interests or personal relationships that could have appeared to influence the work reported in this paper.
